# Characterizing Gynecological Cancers with the Uncommon dMMR/MSS Phenotype in Lynch Syndrome Patients

**DOI:** 10.3390/jcm15134979

**Published:** 2026-06-26

**Authors:** Georgios Koktsidis, Davide Mauri, Stylianos Elemes, Emmanouil Saloustros, Genovefa Polychronidou, Georgios Lazaridis, Dimitrios Dionysopoulos, Anna Papaioannou, Eleni T. Timotheadou

**Affiliations:** 1 Department of Oncology, “Papageorgiou” Hospital, Aristotle University of Thessaloniki, 56403 Thessaloniki, Greece; dvd.mauri@gmail.com (D.M.); stelemes.md@gmail.com (S.E.); georlaz@yahoo.gr (G.L.); dimitris.dionysopoulos@gmail.com (D.D.); annapriestjohn96@gmail.com (A.P.); timotheadou@gmail.com (E.T.T.); 2 Division of Oncology, Faculty of Medicine, School of Health Sciences, University of Thessaly, 41500 Larissa, Greece; esaloustros@med.uth.gr; 3 First Department of Clinical Oncology, Theagenio Cancer Hospital, 54007 Thessaloniki, Greece; vefap@yahoo.gr

**Keywords:** dMMR/MSS, DNA mismatch repair (MMR), immune checkpoint inhibitors (ICI), Lynch syndrome, microsatellite stability (MSS)

## Abstract

**Background/Objectives:** Lynch syndrome is an inherited autosomal dominant disease that predisposes to a broad spectrum of malignancies. This condition is driven by a germline pathogenic variant in one or more of the mismatch repair (MMR) genes (MLH1, MSH2, MSH6, PMS2, EPCAM) that results in genomic microsatellite instability (MSI-high). However, microsatellite-stable (MSS) tumors in Lynch syndrome patients may occur, but the relevant literature is scant. Our aim is to systematically identify, organize, and characterize the comprehensive available literature for Lynch syndrome-associated gynecological cancers (vulvar, vaginal, cervical, ovarian, endometrial) exhibiting a dMMR/MSS phenotype. **Methods:** Systematic review of the literature. Three medical databases, major related conferences and relevant oncology journals were scrutinized for relative evidence. **Results:** Eleven reports were identified comprising 2351 patients, 774 had confirmed Lynch syndrome (LS), of whom MSI results were available for 299 patients. In total, 130 patients developed LS-associated gynecological cancers, for whom MSI results were available. Among them, 36 tumors (27.7%) exhibited a dMMR/MSS molecular phenotype (23 endometrial, 12 ovarian, one synchronous endometrial–ovarian). No data for vulvar, vaginal, or cervical cancers were found. For dMMR/MSS endometrial cancers, the mean age at diagnosis was 50.5 years (range 36–62). dMMR/MSS tumors arose predominantly in MSH6 and, to a lesser extent, PMS2 carriers. No data on selective response to immune checkpoint inhibitors treatment were available. **Conclusions:** Lynch syndrome-associated gynecological cancers with dMMR/MSS phenotype are strongly underrepresented in the literature. Endometrial and ovarian cancers with dMMR/MSS phenotype are early onset and arise in a small proportion of patients. Better characterization is demanding to unearth whether response to immunotherapy treatment vary across Lynch dMMR/MSS and dMMR/MSI-High phenotypes.

## 1. Introduction

Lynch syndrome (LS) (previously known as Hereditary Nonpolyposis Colorectal Cancer—HNPCC) is an inherited multisystemic cancer predisposition syndrome. It is an autosomal dominant condition defined by the presence of a constitutional pathogenic variant in one or more of the mismatch repair (MMR) genes MLH1, MSH2, MSH6 and PMS2, or in deletions of the EPCAM gene, which regulates gene MSH2 expression. It is now well known that LS is multisystemic cancer predisposition syndrome, which among other malignancies, increases the lifetime risk of gynecological cancers, particularly endometrial and ovarian cancer. It accounts for approximately 3% of all endometrial carcinomas [1] and 2–3% of all ovarian cancers [2]. Malignancies in patients with LS are generally characterized by DNA mismatch repair deficiency (dMMR) and high microsatellite instabillity (MSI-high), which in turn creates a milieu of somatic mutations of target genes. These mutations are subsequently presented by HLA class I receptors as neoantigens, which engage and activate the immune system. As such, tumors with dMMR/MSI-H molecular phenotype promote antitumor immune cells recognition and have increased Tumor Infiltrating Lymphocytes (TILs), thus making these tumors more likely to respond to anti-PD-1/PD-L1 immunotherapy [3,4,5]. In parallel with advances in the understanding of LS-related tumor biology, recent studies have further emphasized the molecular complexity of gynecological cancers, including non-coding RNA-driven oncogenic mechanisms, tumor microenvironment-mediated immune regulation, and emerging targeted therapeutic strategies [6,7,8]. These data collectively support the notion that gynecological malignancies comprise biologically diverse entities whose behavior may not be fully captured by conventional clinicopathological classification alone. Although such evidence does not specifically address Lynch syndrome-associated gynecological tumors, it reinforces the need for more refined molecular characterization of this uncommon subgroup. Specifically with regard to Lynch syndrome, little is known about the population of patients with Lynch syndrome and gynecological cancer presenting with dMMR/MSS phenotype and its related implication for immunotherapy.

To address this gap, we performed a systematic review of the existing literature to scrutinize the comprehensive cumulative available evidence and depict clinicopathological features of patients with confirmed LS who develop gynecological cancers with a dMMR/MSS phenotype.

## 2. Material and Methods

### 2.1. Search Strategy

A literature search was performed by two independent reviewers (G.K., S.E.) in three medical libraries PubMed/MEDLINE, Scopus and Embase (Last Search: 31 October 2025), using as algorithm base the search string (lynch AND mss) AND (cancer OR adenocarcinoma OR tumor OR neoplasm OR sarcoma) and ran six site-specific permutations by substituting the anatomic block with the terms:

1. (vulvar OR vaginal)
2. (cervix OR cervical)
3. (ovary OR ovarian)
4. (endometrial OR endometrium)
5. (gynecological)
6. (adnexal)

The selected anatomic descriptors were chosen to cover the principal gynecological sites relevant to the scope of the review, namely vulvar/vaginal, cervical, ovarian and endometrial disease, while also including the broader descriptors “gynecological” and “adnexal” to capture reports that might not be indexed under a single organ-specific term.

In case of disagreement between independent searches, an independent Gynecological Cancer Expert (E.T.) supervised the data.

To ensure that electronic searches would not miss reports of eligible studies, two independent reviewers also searched for the years 2020 through 2025 in several oncology major journals: New England Journal of Medicine, Lancet, Lancet Oncology, Journal of Clinical Oncology, Annals of Oncology, ESMO Open and the ASCO post (G.K., G.L.) [9].

The reference list of retrieved papers was further screened for additional publications to minimize publication bias (G.K., A.P.).

Considering that recent trials may still be unpublished, we also reviewed the abstract books and presentation archives from major meetings of the American Society of Clinical Oncology and the European Society of Clinical Oncology held between 2020 and 2025, to identify any additional reports presenting final comparative data eligible for inclusion in the review (American Society of Clinical Oncology ASCO, European Society of Medical Oncology ESMO, ESMO Gynecological Cancers). Earlier meeting abstracts were not included (D.M., S.E.).

The completed PRISMA 2020 checklist is provided in the Supplementary Materials.

### 2.2. Eligibility Criteria

The search topic was dMMR/MSS gynecological cancers among patients with confirmed Lynch syndrome. Studies were eligible regardless of therapy use, treatment with immune checkpoint inhibitors, dose and schedule, or line of treatment. In addition, studies were considered eligible only if MSI results were reported with sufficient detail to permit assignment of tumor-level MSI status in the gynecological cancer subset of patients with confirmed Lynch syndrome, allowing identification of MSS versus MSI-high cases. Potential overlap between reports was assessed by cross-checking the study setting, institution or registry, recruitment period and available clinocopathological characteristics. Whenever multiple reports pertained to overlapping groups of patients, for the analysis of the cumulative available evidence we retained only the report with the largest/most up-to-date data. Data from interim analyses and abstract reports were eligible if no final data were available.

### 2.3. Data Extraction

From each eligible report (G.K., E.S., D.M., G.P.), we recorded the following items: authors’ names; journal and year of publication; country of origin; years of patient enrollment; number of centers involved; number of patients included in the study; number of eligible patients; age at diagnosis; dMMR profile (germline pathogenic variants); MSS and MSI-H phenotypes; and any immunotherapy regimens used.

## 3. Results

### 3.1. Flow of the Systematic Review and Eligible Reports Identified

**Figure 1:**
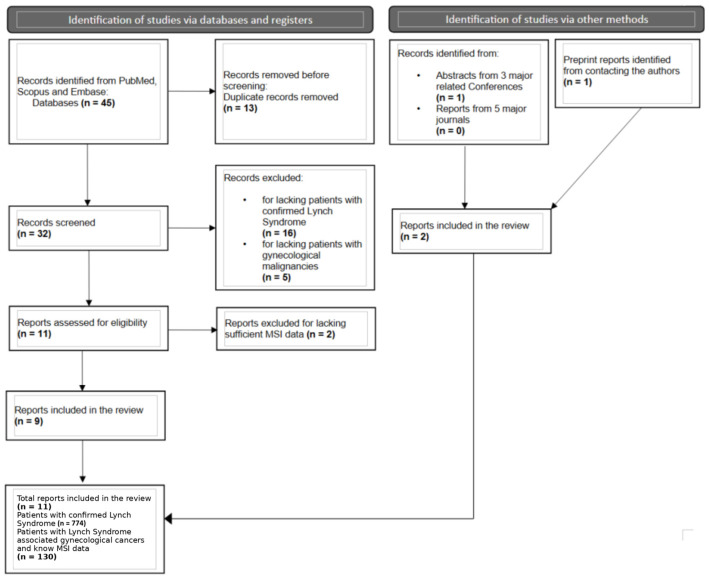
Flow chart of the study.

A total number of 45 articles were initially identified through the systemic searches in PubMed, Embase and Scopus databases. After removing duplicates (*n* = 13), 32 unique records were screened. Of these, sixteen articles were excluded for lacking patients with confirmed germline pathogenic variants in mismatch repair (MMR) genes required to confirm Lynch syndrome. An additional five articles were excluded because they did not include any patients with gynecological malignancies. Eleven full-text articles were assessed for eligibility, of which two were excluded for lacking sufficiently detailed MSI data [Figure 1]. Ultimately, nine studies met the inclusion criteria [10,11,12,13,14,15,16,17,18], encompassing 740 patients with confirmed LS, of whom MSI results were available for 265 patients.

Overall, from the systematic search of the medical databases, a total number of 124 cases with gynecological cancer and known MSI status were retrieved, among whom 31 patients with a dMMR/MSS molecular tumor phenotype were identified.

In addition, research in abstracts from three major related conferences (ESMO, ESMO Gynecological Cancers, ASCO) and in records from five major journals (Annals of Oncology, The Lancet, The New England Journal of Medicine, ESMO Open and The ASCO Post) from the past 5 years yielded a single report [19] pertaining to three more patients with LS-associated dMMR/MSS gynecological cancers. Finally, through correspondence with the authors of the aforementioned studies, an additional preprint report was retrieved [20], which included two patients with Lynch syndrome-associated dMMR/MSS gynecological cancers [Figure 1].

### 3.2. Descriptive Analysis

Reported data across the eligible studies were scarce and heterogeneous, and a pooled analysis could not be performed. We therefore use descriptive statistics to summarize the available data. In total, eleven reports comprising 2351 patients were identified. The data originated from registries and cancer centers in the United States [Houston, Texas [10] and Ohio [14]], Spain (Alicante) [11], Japan [Nagaizumi [12] and Sapporo [17]], Denmark [13], United Kingdom (Manchester) [14], the Netherlands [15], China [Chengdu [16] and Beijing [18]], and North Macedonia [19,20].

Of all the patients, 774 had a documented diagnosis of Lynch syndrome (LS), 323 of whom developed LS-associated gynecological cancer. MSI results were available for 130 of these patients. Overall, 36 cases exhibited the dMMR/MSS molecular phenotype, corresponding to 27.7% of the LS-associated gynecological cancer cohort (36/130).

**Endometrial Cancer:** dMMR/MSS pattern was observed in 23 patients with endometrial cancer and 12 patients with ovarian cancer, while one patient was diagnosed with synchronous primary malignant neoplasms of the ovary and endometrium, both characterized by a dMMR/MSS phenotype. Among the 23 patients with endometrial cancer, age at diagnosis was reported for 12 of them, with a range of 36–62 years, a mean age of 50.5 years, and a median age of 50.5 years. Although these data are limited, the mean age at diagnosis of these malignancies is substantially lower than the mean age at diagnosis of endometrial cancer in the general population (64 years) [21]. The patient with synchronous ovarian and endometrial cancer was diagnosed at 45 years of age.

In the corresponding reports, among patients with sporadic endometrial cancer for whom age at diagnosis was available, the pooled mean age at diagnosis was 58.1 years [Bruegl et al.: 61.3 years [10]; Egoavil et al.: 63.3 years [11]; Wang et al.: 53 years [16]; Matsubayashi et al.: 62.5 years [12]; Staninova et al.: 64.3 years [20]], whereas the mean age at diagnosis of endometrial cancer with a typical dMMR/MSI-high profile in patients with Lynch syndrome was 50.6 years [Bruegl et al.: 50.8 years; Egoavil et al.: 49 years; Wang et al.: 54 years; Matsubayashi et al.: 51.5 years; Staninova et al.: 46.7 years; van der Werf et al.: 55.5 years [15]].

With respect to the histologic subtype of endometrial cancers, detailed data for patients with confirmed LS were not consistently reported, apart from the study by Egoavil et al. [11], in which seven of eight LS patients (87.5%) had an endometrioid subtype. In the overall population of patients for whom histologic information was available, the vast majority were diagnosed with endometrioid endometrial carcinoma (Bruegl et al.: 74.2%; Egoavil et al.: 82.9%; Wang et al.: 83.8%).

Of the 23 patients with dMMR/MSS endometrial cancer, 19 were genetically characterized by a pathogenic MSH6 variant, two by a pathogenic PMS2 variant, and two by concurrent pathogenic variants in both MSH6 and MSH2. The patient with synchronous ovarian and endometrial cancer harbored a pathogenic MSH6 variant. The findings of our review are summarized in Table 1. No data related to treatment with checkpoint inhibitor immunotherapy were available in any eligible report.

**Ovarian Cancer:** Age at diagnosis was not available for the 12 patients with ovarian cancer. Among these patients with LS-associated ovarian cancer [10], 55.6% had endometrioid morphology, 22.2% clear cell, 11.1% mucinous, and 11.1% serous morphology. Histologic subtype was not reported specifically for the dMMR/MSS cases. No data related to treatment with checkpoint inhibitors immunotherapy were available in any eligible report.

**Vulvar, vaginal, cervical or adnexal cancers:** No reports on dMMR/MSS cancers were identified in the literature searches.

## 4. Discussion

This study represents, to our knowledge, the first systematic effort to characterize dMMR/MSS gynecological tumors in patients with germline confirmed Lynch syndrome, drawing on data from multiple registries and cancer centers across Europe, North America, and Asia.

With the sole exception of the article by Egoavil et al. [11], which was published in 2013, and that by Bruegl et al. [10], published in 2017, all the data we collected were published during the last four years. This timing strongly suggests that this particular population of patients with a documented diagnosis of LS and dMMR/MSS gynecological tumors has only very recently begun to be recorded and therefore remains significantly underestimated.

Despite the limited literature and the very recent recognition of this patient population, the available descriptive data suggest that patients with LS and dMMR/MSS gynecological tumors are generally early onset cancers, as they are diagnosed at a much younger age compared with the general population, with their age at diagnosis being closest to the mean age at diagnosis of typical LS-associated tumors with a dMMR/MSI-high molecular phenotype. This phenotypic pattern suggests that deficiency in one or more of the MMR genes is fundamental to the process of oncogenesis, even when microsatellite regions remain stable. Therefore, gynecological cancers with a dMMR/MSS molecular profile, although clearly limited in number, may belong to the spectrum of Lynch syndrome and should not be uncritically regarded as sporadic cancers.

Unexpectedly, considering that we are in the immunotherapy era, and since the potential response to treatment may vary across LS-associated dMMR/MSS and LS-associated dMMR/MSI-H phenotypes, no data on the use of immune checkpoint inhibitor therapy in patients with LS-associated Dmmr/mss gynecological cancers were available in any eligible report.

From a mechanistic standpoint, we know that MMR gene deficiency is functionally associated with a multitude of genetic defects and gene-level errors, which cause an increase in the expression of tumor neoantigens. The genetic predisposition of patients with Lynch syndrome to develop microsatellite instability-high tumors, with the consequent increase in tumor neoantigen burden, has been shown to be associated with the efficacy of immune checkpoint inhibitors in this patient population. This phenomenon has been documented both biologically and clinically in gynecological malignancies as well [22]. The finding, however, of microsatellite stability in tumors with MMR gene deficiency may indicate an intermediate biological state with an undefined burden of mutations and neoantigens and reduced immunogenicity, which makes it difficult to predict the response of these patients to immune checkpoint inhibitors. Indeed, previous studies on this subject have shown a limited role of immunotherapy in this specific category of patients [23]. As such, the position of dMMR/MSS tumors in the framework of the classical rationale of immunotherapy needs further investigation to be better characterized.

Important limitations of our review must be acknowledged. Our study is purely retrospective and therefore particularly heterogeneous regarding patient selection and the technical characteristics of each study. Apart from the notably small number of patients that we recorded, we also collected a limited amount of data regarding age at diagnosis, histological characteristics of the tumors, and stage of each disease. These gaps were obviously particularly important for the complete characterization of the population we describe in the present work. In addition, no central pathology review was performed, and all histopathological and pathology-related variables were extracted from the original published reports, which represents a methodological limitation and may have further contributed to inter-study heterogeneity. Furthermore, none of the studies included in the present systematic review reported treatment details and responses, and therefore no reliable conclusions can be drawn regarding the benefit of immunotherapy in this patient population.

## 5. Conclusions

In conclusion, and despite these limitations, this systematic review constitutes a first attempt to highlight this specific population of patients with LS and associated gynecological cancers with a dMMR/MSS phenotype. These data should motivate prospective, standardized studies to clarify the biology and optimal treatment of this under-recognized subgroup.

## Figures and Tables

**Table 1:** Clinicopathologic and genetic characteristics of Lynch syndrome-associated gynecological cancers with the dMMR/MSS phenotype.

Patients’ Demographics	
Total Patients scrutinized	2351
LS Patients	774
LS associated gynecological cancers with known MSI data	130
dMMR/MSS gynecological cancers in LS patients	23 Endometrial
	12 Ovarian
	1 synchronous Endometrial + Ovarian
**Endometrial Cancer**	
Mean age at diagnosis of dMMR/MSS endometrial cancers (years)	50.5 (range 36–62; n = 12)
Mean age at diagnosis of sporadic endometrial cancers (years)	58.1
Mean age at diagnosis of dMMR/MSI-high endometrial cancers (years)	50.6
Germline variants of dMMR/MSS	19 MSH6
	2 PMS2
	2 MSH + MSH2
**Ovarian Cancer**	
Mean age at diagnosis of dMMR/MSS ovarian cancers (years)	NA
Germline variants of dMMR/MSS	NA

dMMR: MMR deficiency, LS: Lynch syndrome, MSS: microsatellite stable, NA: not available.

## Data Availability

No new data were created or analyzed in this study.

## Supplementary Materials

The following supporting information can be downloaded at: https://www.mdpi.com/article/10.3390/jcm15134979/s1, PRISMA 2020 Checklist [24].

## Abbreviations

The following abbreviations are used in this manuscript:

ASCO	American Society of Clinical Oncology
dMMR	MMR Gene Deficiency
ESMO	European Society For Medical Oncology
LS	Lynch Syndrome
MSI-high	Microsatellite Instable
MSS	Microsatellite Stable
MMR	Mismatch Repair
